# Ancient DNA from Hunter-Gatherer and Farmer Groups from Northern Spain Supports a Random Dispersion Model for the Neolithic Expansion into Europe

**DOI:** 10.1371/journal.pone.0034417

**Published:** 2012-04-25

**Authors:** Montserrat Hervella, Neskuts Izagirre, Santos Alonso, Rosa Fregel, Antonio Alonso, Vicente M. Cabrera, Concepción de la Rúa

**Affiliations:** 1 Department of Genetics, Physical Anthropology and Animal Physiology, University of the Basque Country, Bizkaia, Spain; 2 Department of Genetics, University of La Laguna, La Laguna, Santa Cruz de Tenerife, Spain; 3 Department of Biology, National Institute of Toxicology and Forensic Sciences, Madrid, Spain; University of York, United Kingdom

## Abstract

**Background/Principal Findings:**

The phenomenon of Neolithisation refers to the transition of prehistoric populations from a hunter-gatherer to an agro-pastoralist lifestyle. Traditionally, the spread of an agro-pastoralist economy into Europe has been framed within a dichotomy based either on an acculturation phenomenon or on a demic diffusion. However, the nature and speed of this transition is a matter of continuing scientific debate in archaeology, anthropology, and human population genetics. In the present study, we have analyzed the mitochondrial DNA diversity in hunter-gatherers and first farmers from Northern Spain, in relation to the debate surrounding the phenomenon of Neolithisation in Europe.

**Methodology/Significance:**

Analysis of mitochondrial DNA was carried out on 54 individuals from Upper Paleolithic and Early Neolithic, which were recovered from nine archaeological sites from Northern Spain (Basque Country, Navarre and Cantabria). In addition, to take all necessary precautions to avoid contamination, different authentication criteria were applied in this study, including: DNA quantification, cloning, duplication (51% of the samples) and replication of the results (43% of the samples) by two independent laboratories. Statistical and multivariate analyses of the mitochondrial variability suggest that the genetic influence of Neolithisation did not spread uniformly throughout Europe, producing heterogeneous genetic consequences in different geographical regions, rejecting the traditional models that explain the Neolithisation in Europe.

**Conclusion:**

The differences detected in the mitochondrial DNA lineages of Neolithic groups studied so far (including these ones of this study) suggest different genetic impact of Neolithic in Central Europe, Mediterranean Europe and the Cantabrian fringe. The genetic data obtained in this study provide support for a random dispersion model for Neolithic farmers. This random dispersion had a different impact on the various geographic regions, and thus contradicts the more simplistic total acculturation and replacement models proposed so far to explain Neolithisation.

## Introduction

The phenomenon of Neolithisation refers to the transition from a hunter-gatherer way of life to an agro-pastoralist lifestyle, involving crop farming and livestock herding. There is consensus on the origin of the agro-pastoralist lifestyle associated with the Neolithic in the Near East, from where it spread throughout Europe. Yet, there is no such agreement on the mechanisms and means of transmission of farming to Europe. Traditionally, the spread of crop farming and livestock herding in Europe during the Neolithic has been framed within a dichotomy based either on an acculturation phenomenon or on a demic diffusion process.

The demic diffusion model describes a migratory process based on a population expansion from the Near East into Europe, whose consequence was the assimilation of the genetic pool of the indigenous hunter-gatherer groups by the expanding of the farming community [1]–[6]. On the other hand, the acculturation model posits that this transition occurred through the adoption of the agro-pastoralist system by local indigenous groups, without receiving any genetic input [7]–[9]. Between these two models there are others that suggest a varying intensity of the genetic impact from the Neolithic farming communities that spread throughout Europe from the Near East [10], [11].

The analysis of the genetic composition of present-day populations in Europe and the Near East, has tried to establish the origin of their extant genetic variability. Based on classical genetic markers, Ammerman and Cavalli-Sforza [1], proposed the wave-of-advance model, whereby the demic diffusion from the Near East towards Europe contributed to the genetic composition of present-day populations. Nevertheless, based on modern European patterns of mitochondrial diversity, Richards et al. [9] argued that this mitochondrial diversity had a predominantly Paleolithic origin, with a small Neolithic contribution (12%), which would favour the cultural diffusion model. Subsequent studies applying new methodologies have allowed quantifying the contribution of Neolithic farmers to the genetic pool of present-day European populations at 23% [12], [13].

Several studies on the variability of the non-recombining region of the Y chromosome (NRY) have detected the presence of a southeast-northwest gradient in Eurasia, which has been interpreted as the genetic fingerprint of Neolithic expansion [14], [15]. A recent analysis of the variability of the Y chromosome in more than 2,500 samples taken from present-day European population revealed that, the diversity of haplogroup R1b1b2 (the most common one in Europe) is best explained by the spread from a single source in the Near East via Anatolia during the Neolithic [16]. This proposal contradicts prior studies, which consider this haplogroup to be a marker of the Mesolithic re-expansion from the glacial refuge in the Franco-Cantabrian region, the Balkans and the Alps [14], [15].

The analysis of the DNA recovered from ancient human remains has highlighted a more complex pattern than the one revealed by the studies of contemporary populations [17]–[22] (amongst others). Studies hitherto conducted on ancient DNA (aDNA) from European Neolithic populations indicate two routes to explain the Neolithic dispersion to Europe: one, supporting an acculturation model that followed a route of dispersion through the North-Central Europe [8], [23], and a second one, supporting a demic diffusion model through a Mediterranean route [24], [25].

Ancient DNA studies of hunter-gatherers from Scandinavia and Central Europe have pointed to a genetic discontinuity between hunter-gatherer and Neolithic populations in these two geographic regions [26], [27]. The analysis of mtDNA in the first Neolithic farmers of Central Europe (7.5–7 kya) showed a high frequency of haplogroup N1a (15%), which is rarely found in present-day populations (0.2%) [8], [23]. On the other hand, these first farmers showed a genetic affinity with the present-day populations from the Near East and Anatolia, supporting a major genetic input from this area during the advent of farming in Europe [23].

The mtDNA analysis from the Neolithic site of the Iberian Mediterranean region named, “*Camí de Can Grau*” in Catalonia (5.5–5 kya), [24] has provided results that are different to those published by Haak et al. [8], [23]. The absence of haplogroup N1a and the presence of mitochondrial lineages typical of present-day populations from this European Mediterranean area, led Sampietro et al. [24] to propose a demic diffusion only in Mediterranean area of Europe.

Likewise, the combined analysis of the mtDNA and NRY variability in 29 individuals from a French Mediterranean region, named “*Cave I of Treilles*” in Aveyron, (5 kya), [25] reaffirmed the aforementioned proposal, based mainly on the absence of the mitochondrial haplogoup N1a and the R1b of the NRY, considered Neolithic dispersion markers through Central Europe [8], [16], [23].

**Figure 1:**
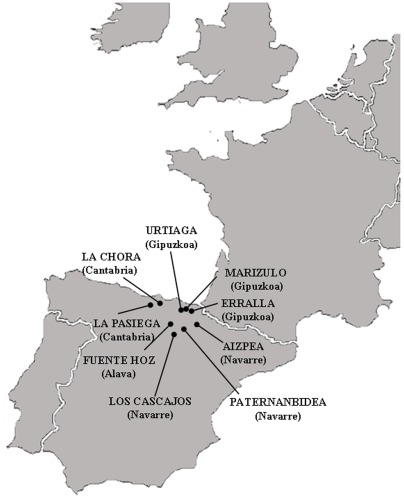
Geographic location of the ancient human remains analysed in the present study. All sites are located in the North of the Iberian Peninsula.

In view of the debate surrounding the phenomenon of Neolithisation in Europe, we have carried out a genetic analysis of Paleolithic hunter-gatherer and Neolithic samples recovered from nine sites in the North of the Iberian Peninsula (Basque Country, Navarre and Cantabria), whose chronologies date from the Upper Paleolithic through to the Bronze Age (Figure 1 and Table 1). The aim of this study is to explain the Neolithisation process along the Iberian Cantabrian fringe within the context of European populations.

**Table 1:** Chronology, subsistence pattern and geographical origin of the prehistoric samples analysed in the present study.

SITES	NOMENCLATURE	SUBSISTENCE PATTERN	CHRONOLOGY	MNI	N	REFERENCES
La Pasiega (Cantabria)	PS	Hunter-Gathering(**HG_CANT**)	Upper Paleolithic (Magdalenian)	1	1	[44]
La Chora (Cantabria)	CH	Hunter-Gathering (**HG_CANT**)	Upper Paleolithic (Magdalenian)	1	1	[45]
Erralla (Gipuzkoa)	ERR	Hunter-Gathering (**HG_CANT**)	Upper Paleolithic (Magdalenian) (12,310 BP)	1	1	[46]
Aizpea (Navarre)	AZ	Hunter-Gathering (**HG_CANT**)	Mesolithic (6,600±65 BP)	1	1	[47]
Los Cascajos (Navarre)	CAS	Early Neolithic farming (**NEO_NAVARRE**)	Neolithic (6,185–5,185 BP)	32	27	[48]
Paternanbidea (Navarre)	PAT	Early Neolithic farming (**NEO_NAVARRE**)	Neolithic (6,090−5,960 BP)	9	9	[49]
Marizulo (Gipuzkoa)	MZ	Neolithic farming	Neolithic (5,285 BP)	1	1	[50]–[51]
Fuente Hoz (Álava)	FH	Neolithic farming	Neolithic (5,240–5,160 BP)	6	6	[52]
Urtiaga (Gipuzkoa)	URT	Wild and domestic resources	Bronze Age (3,475−3,430 BP)	2	2	[53]

Minimal number of individuals at each sites (MNI), number of individuals analysed (N).

## Results

### Authenticity of the results

We have successfully analyzed 49 out of 54 individuals recovered from the nine prehistoric sites analyzed in the present study; four sites correspond to hunter-gatherer groups (La Chora, La Pasiega, Erralla and Aizpea), another four to the Neolithic period (Los Cascajos, Paternanbidea, Marizulo and Fuente Hoz) and the last one to the Bronze Age (Urtiaga). Out of these 49 individuals, 30 (61% of the individuals) were analysed in duplicate in our laboratory; in addition, a third sample from each of 22 individuals (44% of the individuals) was replicated at an independent laboratory (some in the University of La Laguna and others in the INTCF of Madrid, Spain).

The number of molecular targets (mtDNA copy number) was quantified for each extract by means of quantitative real-time PCR (qPCR). The results showed that the number of molecules per μl in the extracts ranged between 100–12,000 (Table S1). These values are within the limits proposed for reproducibility in aDNA studies [28].

In addition, a total of 69 PCR products from 49 individuals were cloned. A minimum of ten clones per PCR product were amplified and sequenced. The results were used to determine the coincidence between the consensus sequence obtained from the clones and the sequence obtained by direct sequencing of the initial PCR product. A mean of 4.20 mutations per fragment cloned (∼100 bp) were rejected, as these mutations were found in single clones, possibly as consequence of post-mortem damage to the aDNA molecules (Table S2).

In order to identify any possible contamination that might have occurred in the different stages of the laboratory work, at least two extraction controls and several PCR negative controls were included in each amplification. If any contamination was detected, the results obtained were discarded.

Finally, the mtDNA HVR-I segment from the researchers and archaeologists that handled the samples was sequenced in order to rule out any possible contamination (Table S3). A comparison between the haplotypes of the researchers/archaeologists and those obtained in the aDNA samples (Table 2) produced two cases of coincidence, one with the rCRS haplotype, which is the most frequent one in European populations, and the other with haplogoup K (ht18: 92-224-311), which is widely present not only throughout the whole of the Iberian Peninsula [29] and the Canary Islands [30], but also Europe [9], [12], [13] and North Africa [31]. HVR-II sequencing was performed on those prehistoric samples that coincided with the haplotypes of the researchers and/or archaeologists in order to discard a complete coincidence between them.

### Analysis of mtDNA variability in the prehistoric samples

We have successfully analyzed 49 individuals from nine prehistoric sites and obtained 25 different mitochondrial haplotypes belonging to eight mitochondrial haplogroups (H, U, K, J, HV, I, T and X) (Table 2). Haplogroup H is the major one, showing a frequency of 45% in the ancient samples analysed. This figure is similar to that observed in some present-day European populations including the North of the Iberian Peninsula [13], [32]–[34].

The second most frequent haplogroup was haplogroup U (34.7%) (Table 2), being the Neolithic populations of Navarre (Los Cascajos and Paternanbidea) the ones that showed the highest frequencies for this haplogroup (29.6% and 11.1%, respectively). A special mention should be made of sub-haplogroup U5, as it is one of the oldest found in Europe (∼36.9 kya), and has been linked to the colonisation of Europe by anatomically modern *Homo sapiens* (AMHS) in the Early Upper Paleolithic [13], [35]–[37]. The average frequency of U5 in the present study was 12%, considering hunter-gatherer samples (Erralla and Aizpea), Neolithic samples (Los Cascajos and Marizulo) and the Bronze Age sample (Urtiaga) (Table 2).

Haplogroup J exhibited a frequency of 4.08% in the samples analysed, being found in two individuals from Los Cascajos, with two different haplotypes: ht7 and ht12 (Table 2).

Finally, haplogroup K was only found in the Neolithic sites of Navarre (Los Cascajos and Paternanbidea), showing an average frequency of 8.2%. All the other haplogroups (HV, I, T and X) had a frequency of around 2% each, with haplogroups HV and I being only found in Paternanbidea, and haplogroups T and X in Los Cascajos (Table 2).

**Table 2:** Distribution of the frequencies (%) of the mtDNA haplotypes (HT) and haplogroups (HG) obtained in the prehistoric samples from the present work (see nomenclature in Table 1).

SAMPLES	HT	HVS-I-II	N	%	HG	%
CAS-21	ht1	73	1	0.02	H	
CAS-33; CAS-182; CAS-497; PAT-1E3; PAT-2E1; FH-6; URT-1; PS-1	ht2	rCRS	8	0.163	H	
CAS-48, CAS-90, CAS-196	ht3	73-146-263-285-309.1C-310.1T-312-313	3	0.061	H	
CAS-173; CAS-222; CAS-341	ht6	16129	3	0.061	H	
CAS-194; CAS-193S	ht11	16311	2	0.041	H	
PAT-1E4, PAT-4E2	ht16	16209	2	0.041	H3	
PAT-1E5	ht17	16092-16311	1	0.02	H	
FH-3	ht23	16093	1	0.02	H	
CH-1	ht24	16093-16362	1	0.02	H6	44.9
CAS-70; CAS-216; CAS-254; CAS-258; PAT-1E1; FH-1; FH-4; FH-5	ht4	73-146-263-285-309.1-310.1-312-313	8	0.163	U	
CAS-148	ht5	16278-16311	1	0.02	U	
CAS-204	ht13	16270-16311	1	0.02	U5	
ERR-1; MZ-1	ht21	16270	2	0.041	U5	
FH-2; URT-2;	ht22	16192-16270	2	0.041	U5a	
CAS-183	ht10	16163	1	0.02	U	
CAS-517	ht15	16365	1	0.02	U	
AIZ-1	ht25	16051-16093-16189-16192-1270	1	0.02	U5b1	34.69
CAS-181; CAS-191; CAS-202	ht9	16224-16311	3	0.061	K1a	
PAT-2E2	ht18	16092-16224-16311	1	0.02	K	8.163
CAS-179	ht7	16069-16126-16195	1	0.02	J	
CAS-203	ht12	16069-16129	1	0.02	J	4.082
PAT-3E2	ht19	16311	1	0.02	HV	2.041
PAT-4E1	ht20	16129-16233	1	0.02	I	2.041
CAS-180	ht8	16126-16294-16296	1	0.02	T2	2.041
CAS-257	ht14	16183-16189-16233-16278	1	0.02	X	2.041

rCRS: revised Cambridge Reference Sequence

### Statistical and multivariate analysis of the mitochondrial variability of prehistoric populations

The prehistoric populations analysed in this study (Table 1) were compared with other present-day populations from the Iberian Peninsula, Europe and the Near East, as well as with those prehistoric populations reported in the literature (Table S4).

**Figure 2:**
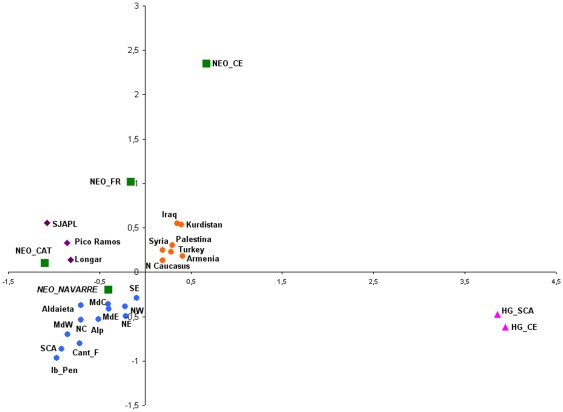
Multidimensional Scaling (MDS), considering the Fst genetic differences calculated according to the distribution of the mtDNA haplogroup frequencies of different populations; Chalcolithics in the Basque Country (purple), Neolithics (green), present-day Near East and northern Caucasus (orange) and Europeans (blue). Abbreviations for populations. Hunter-gatherer groups: Scandinavia (HG_SCA), Central Europe (HG_CE) and Cantabrian fringe (HG_CANT: La Chora, La Pasiega, Aizpea and Erralla). **Neolithic populations:** Catalonia (NEO_CAT), Central Europe (NEO_CE), France (NEO_FR) and Navarre (Los Cascajos and Paternanbidea) (NEO_NAVARRE). **Chalcolithic** **populations in the Basque Country:** Longar, SJaPL and Pico Ramos. **Present-day populations in Europe:** Eastern Mediterranean (MdE), Central Mediterranean (MdC), Western Mediterranean (MdW), Northeast Europe (NE), North-Central Europe (NC), Northwest Europe (NW), Southeast Europe (SE), Scandinavia (SCA), Alps (ALP), Iberian Peninsula (Ib_Pen), and Cantabrian Fringe populations (Cant_F) (that includes the Basque Country)

In order to evaluate the differences between the populations compared, genetic distances have been calculated using the *F_ST_* statistic (Table S5). The three groups of hunter-gatherers considered in this analysis (from Scandinavia, Central Europe and the Cantabrian fringe on the Iberian Peninsula) did not show statistically significant differences between one another. However, the hunter-gatherers from Scandinavia (hg_sca) and those from Central Europe (hg_ce) showed statistically significant differences with other Neolithic, Chalcolithic and present-day populations. The hunter-gatherer group in the Cantabrian fringe (hg_cant) did not show statistically significant differences with any other populations in the comparison, due probably to its small sample size (n = 4), for this reason it was not included in the MDS analysis (Figure 2).

The Neolithic populations from Catalonia (neo_cat), France (neo_fr) and Navarre (neo_cas and neo_pat), did not show statistically significant differences either between each other or with present-day populations. However, the Neolithic population from Central Europe (neo_ce) showed statistically significant differences with the Neolithic populations from Navarre (neo_cas and neo_pat), as well as with present-day populations in both Europe and the Near East (Table S5).

The samples from Los Cascajos and Paternanbidea (neo_navarre) were merged as they did not show statistically significant differences (Table S5) and because the sites were very close in terms of chronology, culture and location (Figure 1).

Concerning the Chalcolithic populations in the Basque Country (San Juan ante Portam Latinam (SJaPL), Pico Ramos and Longar), they did not show statistically significant differences with any of the populations in the comparison set (except with hunter-gatherer groups) (Table S5).

Regarding present-day populations, statistically significant differences were apparent between those in the Near East and those in Europe, indicating a genetic differentiation between these two geographic regions (Table S5).

A Multidimensional Scaling (MDS) analysis was carried out providing a two-dimensional view of the *F_ST_* distance matrix (Figure 2). This analysis showed an RSQ of 0.980 and a stress of 0.0260. The results of the MDS clarified some of the results obtained in previous *F_ST_* pair-wise comparisons (Table S5) and in Principal Component Analysis (PCA) (data not shown), as it is the difference between the hunter-gatherers (hg_sca and hg_ce) and the rest of populations. The hunter-gatherer samples showed the highest frequency values for haplogroup U (50%–80%) than any other present-day population (this haplogroup occupied one end of the first axis in PCA, data not shown). The Neolithic groups are heterogeneous, setting the Neolithics of Central Europe (neo_ce) apart from the rest of Neolithic groups (neo_fr, neo_cat, and neo_navarre). This is because they showed a high frequency for haplogroup N1a, that is absent in other Neolithic populations. The Neolithic sample of France (neo_fr) is closer to present-day populations in the Near East, because it shows similar frequencies for haplogroups TX, W, J, H and U (the ones with the highest correlation for the first axis in the PCA, data not shown). However, the Neolithic populations from the Iberian Peninsula (neo_cat and neo_navarre) are located between the variability of present-day European populations and those in the Near East. Likewise, the Chalcolithic populations in the Basque Country (Longar, SJaPL and Pico Ramos) occupied a similar position to the Neolithic groups of the Iberian Peninsula (Figure 2).

### Phylogenetic analysis of ancient samples

**Figure 3:**
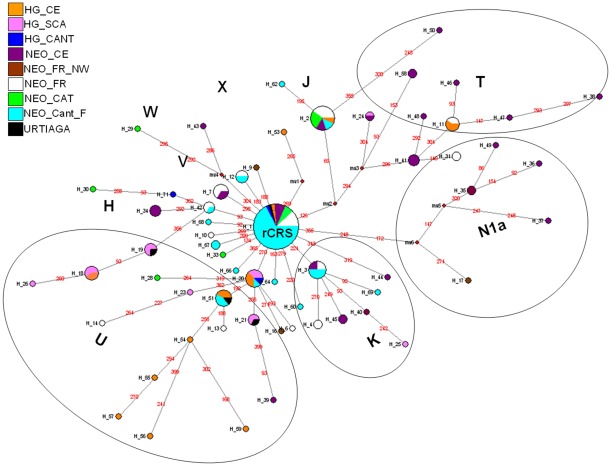
Median Joining Network of European Paleolithic, Neolithic and Bronze Age sequences. Data encompass mtDNA HVR-I (nps 15999-16399). **Hunter-gatherer groups**: Scandinavia (HG_SCA) in pink, Central Europe (HG_CE) in orange, and the Cantabrian fringe (HG_CANT: La Chora, La Pasiega, Aizpea and Erralla) in blue. **Neolithic populations**: Catalonia (NEO_CAT) in green, Central Europe (NEO_CE) in purple, Northwest France (NEO_FR_NW) in brown, South of France (NEO_FR) in white, and the Cantabrian fringe (NEO_Cant_F: Los Cascajos, Paternanbidea, Fuente Hoz and Marizulo) in light blue. Urtiaga (Bronze Age) in black.

We have constructed a Median Joining Network (MJN) (Figure 3) with the HVR-I sequences of the ancient samples obtained in this study, together with a set of sequences of European hunter-gatherer and farming individuals published to date (Table S4). Given the high mutation rate of HVR-I, we applied the substitution rates obtained by Meyer et al. [38], [39] to establish varying mutational weights ranging from 0 to10. The resulting MJN is shown in Figure 3, where the central node is represented by the rCRS, which is shared by all the population groups included in the analysis.

The hunter-gatherer groups in Scandinavia (pink), Central Europe (orange) and Cantabrian fringe (dark blue) share a high number of haplotypes, located in the nodes of the network corresponding to haplogroup U. This figure confirmed the close phylogenetic relationship between the three groups which show the same subsistence pattern (Figure 3).

The sequences corresponding to haplogroup N1a have been found only in the Neolithic groups in Central Europe (purple) and in one individual of a Neolithic site in northwest France (Prissè-la-Chorrière, Deux-Sèvres) (brown), where only three individuals were recovered [40]. This indicates that haplogroup N1a, which has a high frequency in the first Neolithic farmers in Central Europe, is also present in northwest France associated to a late Neolithic group (Figure 3).

Furthermore, we have found shared haplotypes for the Neolithic groups from the Cantabrian fringe (blue), the French Mediterranean area (white) and Catalonia (green). Moreover, we have also found relationship between these three Neolithic groups and the hunter-gatherers from the Iberian Peninsula (dark blue) and Europe (pink and orange). This indicates the presence of shared haplotypes between hunter-gatherers and Neolithic groups from different regions and timeframes (Figure 3).

## Discussion

We have carried out the analysis of the mtDNA variability of a sample of 49 (out of 54) individuals recovered from nine archaeological sites in the north of the Iberian Peninsula (Navarre, Basque Country and Cantabria), with a chronological range from the Upper Paleolithic to the Bronze Age (Table 1). Our goal was to evaluate the model of transition from hunter-gathering to farming in the Cantabrian fringe.

These 49 individuals provided 25 different mitochondrial haplotypes, which indicates high genetic variability (Table 2). Both Paleolithic and Neolithic individuals and even present-day populations along the Cantabrian fringe, share haplotypes albeit at a different proportion between populations.

The mitochondrial variability found in the prehistoric samples analysed here was classified into eight haplogroups: H, U, K, J, I, HV, T, and X, which are amongst the most frequent mitochondrial haplogroups in Europe (Table 2). Nevertheless, the distribution of these haplogroups is different to that in present-day European populations.

The *F_ST_* test indicated that European hunter-gatherer groups (hg_ce, hg_sca and hg_cant) did not show statistically significant differences between them, but they are significantly different from any population compared, because to the high frequency of haplotypes within the haplogroup U (50%–80%) (Table S5, Figure 3). On the other hand, it was noted some differences within the European Neolithic groups, with the Neolithic group in Central Europe (neo_ce) showing the highest number of statistically significant differences in *F_ST_* test, whereas the Neolithics from France and Catalonia (neo_fr and neo_cat) showed the lowest number of statistically significant differences (Table S5). The differentiation of the Neolithic group in Central Europe (neo_ce) is due largely to its high frequency of haplogroup N1a (15%).

Haplogroup N1a was proposed by Haak et al. [8] as a possible genetic marker for the first Neolithic farmers who lived in Central Europe 7,500 years ago [8], [23]. This haplogroup was also found in one individual recovered from a Neolithic burial site in northwest France, confirming its spread to another geographic region in addition to Central Europe [31] (Figure 3). Nonetheless, haplogroup N1a was absent in the Neolithic groups analysed here (neo_navarre), as well as in other Neolithic samples from the Mediterranean area (neo_cat and neo_fr) [24], [25], which might suggest that the genetic influence of Neolithic groups from the Near East in Central Europe was different to that in the western Mediterranean area. This proposal fits well with the lack of simple patterns detectable in the mtDNA data that are available so far.

Haplogroup J, also proposed as a marker for the spread of Neolithic farmers from Near East [13], in the present study has been found only in the ancient Neolithic groups from Navarre (neo_navarre), with a frequency of 6.25%. This value is the lowest of those described for Neolithic populations studied so far, which showed heterogeneous frequencies (neo_fr: 20%, neo_cat: 18% and neo_ce: 9.5%) [8], [23]–[25]. Regarding those lineages belonging to haplogroup J, their influence was not so important in the sites from Navarre compared to other European regions. This result highlights the complex biological patterns resulting from Neolithisation in contrast with the simpler and more evident cultural patterns.

The differences in the genetic composition of Neolithic groups in Europe (including those analysed here) are also evidenced from their respective frequencies for other mitochondrial haplogroups (H, U, I, K, HV, V, T and X). We would like to highlight the absence of haplogroup V, proposed as a marker of the post-glacial recolonization from Franco-Cantabrian refuge [41]. The 49 prehistoric samples analysed in the present study, have not produced a single individual belonging to haplogroup V, which is consistent with previous aDNA studies carried out in the Basque Country [42]. This may suggest the existence of a sub-structuration in populations from Cantabrian Fringe during Paleolithic period that it is maintained up to the present, given the variation of frequencies of haplogroup V in the present-day population from this geographic region [42], [43].

The MDS analysis revealed the difference between the hunter-gatherers and all the other populations compared, as well as the heterogeneity of European Neolithic groups (neo_ce, neo_fr, neo_cat, neo_navarre) (Figure 2). Moreover, it is highlighted the unique position of Central Europe (neo_ce) in relation to their southern counterparts (neo_fr, neo_cat and neo_navarre). This difference seems to indicate that the human groups associated with the spread of agriculture in the region of Central Europe had a different genetic composition to those that followed a Mediterranean route, which were devoid of haplogroup N1a.

The distances observed between the Neolithic groups from the Mediterranean area (neo_fr, neo_cat) and Navarre (neo_navarre) (Figure 2) are also explained by their different frequencies for certain mitochondrial haplogroups, with haplogroups J, U and H standing out (the ones with the highest correlation for the first two axes of the PCA, date not shown). The distances observed between these Neolithic groups could be due to a different genetic impact of Neolithic farmers from Near East into the indigenous populations in the different regions of Europe. This genetic influence could be greater in southern France (neo_fr) than in the Iberian Peninsula. The sites of Los Cascajos and Paternanbidea (neo_navarre), from Early Neolithic, although they showed a substantial Neolithic cultural influence, they seem to show a lower genetic contribution of female migrants from the original areas where Neolithic first developed (Figure 2).

In this regards, the Chalcolithic populations in the Basque Country, dated towards the end of the fifth millennium (SJaPL, Pico Ramos and Longar), showed similar distances with the Neolithic groups in Catalonia (neo_cat) and in Navarre (neo_navarre) (Figure 2). This may be due to the demographic stabilisation of human groups from the Neolithic onwards, which would have attenuated the genetic variations existing during the Paleolithic and first stages of the Neolithic.

In sum, the differences between Hunter-Gatherer and Neolithic groups in Europe can be attributed to the restructuring of their genetic make-up due to the incoming gene flow from the Near East. The genetic data obtained in this mtDNA study contradicts the total acculturation and replacement models proposed for explaining the phenomenon of Neolithisation. Whereas in Central Europe-Northern France a Neolithic clinal gradient can not be discarded from aDNA data so far, the differences in the mtDNA found between Neolithic sites at the Mediterranean area provide support for a random dispersion model. Maritime colonization, transporting small and different Neolithic groups from the Near East pool could contribute to explain the difference.

## Materials and Methods

This study presents a genetic analysis of 49 out of 54 individuals recovered from nine sites located in the north of the Iberian Peninsula (Basque Country, Navarre and Cantabria). Four of those sites correspond to Paleolithic hunter-gatherer populations: La Chora (Cantabria), La Pasiega (Cantabria), Erralla (Gipuzkoa) and Aizpea (Navarre), another four to the Neolithic period: Los Cascajos (Navarre), Paternanbidea (Navarre), Marizulo (Gipuzkoa) and Fuente Hoz (Alava), and the last one to the Bronze Age: Urtiaga (Gipuzkoa) (Figure 1 and Table 1) [44]–[53].

The Neolithic sites from Navarre (Paternanbidea and Los Cascajos) provide the largest samples in this study and are of considerable importance, as on the one hand, they are dated to the Early Neolithic (6.2–5.2 kya) and, on the other, they are found in association with archaeological evidence of fully Neolithic lifestyle (settlements, agriculture and livestock herding) [48]. All the other sites are exceptional in terms of their antiquity and cover an ample spectrum of the geography along the Cantabrian fringe (Figure 1).

The entire excavation process involved strict precautions designed to avoid contamination. Furthermore, the anthropological remains were immediately removed, without undergoing washing, often still embedded in the sediment, and taken to our laboratory where cleaning was performed in dry conditions. The samples analysed in this paper are mostly dental pieces, which constitute the most isolated system in skeletal remains and, therefore, are less liable to outside contamination.

The processing of the samples in the laboratory involved the application of a series of strict criteria for the authentication of results. We used some of the criteria detailed in [54]–[57], such as DNA quantification, cloning, duplication and replication of the results by two independent laboratories. In addition, the extraction and preparation of the PCR were undertaken in a positive-pressure sterile chamber, physically separated from the laboratory where post-PCR processes were carried out. All the work surfaces were cleaned regularly with sodium hypochlorite and irradiated with UV light. Suitable disposable clothing was worn (lab coat, mask, gloves and cap). Contamination controls were applied in both the extraction and amplification processes. In this study, we did not use racemisation of the ancient samples, as several authors have showed this method does not provide efficient information on the reproducibility of the aDNA results [58].

### Collection, Selection and Preparation of Ancient Samples

We have selected those teeth without caries or deep fissures that might extend into the pulp. Whenever possible, more than one tooth was taken from each individual for duplicate analysis, with the duplicates being analysed in different sessions at the University of the Basque Country (UPV/EHU). In addition, a third sample from some individuals was analysed in independent laboratories (either University of La Laguna or National Institute of Toxicology and Forensic Sciences (INTCF), Spain). When dental pieces could not be obtained, bone tissue from long bones was collected (14 samples).

### Extraction of DNA Using Phenol/Chloroform

In order to eliminate surface contamination, the teeth were subjected to a process of depurination using acids, and the entire surface was irradiated with ultraviolet light [59]. In the case of bone-based extraction, the external part of the bone was cleaned by physical scraping, with a piece of bone being then removed and powdered (Freezer Miller Spex 6770, Edison N.J.). The extraction process followed the protocol described by Hagelberg et al. [60]: the tissue was incubated with stirring for 2 hours at 56°C in a lysis buffer (5 ml) (0.5 M EDTA pH 8.0–8.5; 0.5% SDS; 50 mM Tris HCl pH 8.0; 0.01 mg/ml proteinase K). The DNA was recovered using phenol and chloroform and then concentrated and purified (Centricon-30, Amicon). Each extraction session involved two contamination controls that were applied to the entire process, except no dental or bone tissue was added.

### Analysis of the variability of mtDNA

Sequencing of mtDNA HVR-I, nucleotide positions (nps) 15,998–16,400, and mtDNA HVR-II, nps 16504-429 as per [61], was undertaken in six overlapping fragments, each with a length of approximately 100 bp (base pair). HVR-II sequencing was carried out in samples with no polymorphic positions in HVR-I (Table S6). Similarly, the fragment between primers 8F and 8R (Table S6) was amplified in all samples to determine position 73 of HVR-II. The PCRs were performed in 25 μl of reaction mixture containing 10 mM Tris-HCl pH 8.3; 2 mM of MgCl_2_, 0.1 μM of each dNTP, 0.4 μM of each primer, 5 units of AmpliTaq Gold (Applied Biosystems) and 10 μl of diluted DNA (1 μl of DNA extract in 10 μl of 1 mg/ml BSA). Cycling parameters were 95°C for 10 min; followed by 40 cycles of 95°C for 10 sec, annealing temperature for 30 sec, 72°C for 30 sec; and a final step of 72°C for 10 min. The annealing temperatures of the primers of HVR-I were as follows: 60°C for the A1/A1R primer pair, 58°C for 2F/2R and 4F/4R, 57°C for 1F/1R and 55°C for 3F/3R and 5F/5R, the primer sequences are listed in [62]. The sequence and annealing temperatures of the HVR-II primers are shown in Table S6. The amplification of each fragment was undertaken in independent PCRs and each fragment was amplified and sequenced twice from two independent DNA extract. In the case of positive amplification and the absence of contamination, the amplifications were purified by ExoSAP-IT (USB Corporation), with subsequent sequencing in an ABI310 automatic sequencer using chemistry based on dRhodamine. The results obtained were edited with BioEdit software (http://www.mbio.ncsu.edu/BioEdit/bioedit.html) and the sequences were aligned manually.

In order to classify the mitochondrial variability of the individuals analyzed in this study, we proceeded to amplify 11 markers, which are required for defining the 10 Caucasian haplogroups described [63]. The protocol and primers are described in [17], [20], [42]. The digestion patterns were verified using a fragment Bioanalyzer (Agilent Technologies).

### Authentication Methods

In addition to the precautions taken to avoid contamination, other authentication criteria such as quantification, cloning and sequencing were applied.

#### Duplication

61% of individuals were analyzed in duplicated at different times and by different researchers at the University of the Basque Country (UPV/EHU).

#### Replication

The HVR-I of a sub-sample of 21 individuals (43% of the samples) was replicated; almost all of them at the University of La Laguna (Tenerife, Spain) (20 individuals) and the remaining one at the INTCF (Madrid, Spain).

#### Quantification of target DNA

We have quantified the target DNA analysing by quantitative real-time PCR (qPCR), a PCR fragment of 113 bp length of HVR-I, by means of SYBR Green. The methodology is a modification of the one described in [64]. A segment of 113 bp of HVR-I was amplified using primers 5′-CACCATTAGCACCCAAAGCT-3′ and 5′-ACATAGCGGTTGTTGATGGG-3′ [64] in a final volume of 30 μl containing: 2×Power SYBR Green Master Mix (Applied Biosystem), 5 mM of each primer and 10 μl of DNA (dilution 1/10 BSA). Cycling conditions were 1 cycle at 95°C for 10 min, 40–45 cycles at 95 °C for 15 sec and 60°C for 1 min, and a denaturation cycle of 60°C to 96°C. Four replicates were performed for each sample. The standard curve was drawn using eight serial dilutions of a fragment of 405 bp length of the HVR-I, which included the fragment to be quantified. There were four replicates for each dilution obtaining typical values of efficiency (101%) and r^2^ (0.988). In each qPCR, we included the corresponding extraction and amplification controls.

#### Cloning

In order to detect possible heterogeneities in the PCR products that may correspond to either post-mortem damage and/or mixed contamination, a fragment of HVR I was cloned in a sub-sample of 10 individuals by means of the TOPO TA Cloning® Kit (Invitrogen). Linkage to the vector pCR®2.1-TOPO® and chemical transformation of the cells TOP10F' (One Shot® *E. coli*) were performed following the supplier's instructions. After culturing the cells overnight at 37°C in a selective environment, 10 blank cultures were collected and each one cultured in a non-selective environment. Five μl of purified product (using a QIAprep Spin Mini prep Kit, QIAGEN) of each clone were used for the sequencing reaction.

We determined the HVR-I and HVR-II sequence of the mtDNA of the researchers and archaeologists who handled the samples in order to discard possible contamination (Table S3).

### Statistical Analysis

Genetic diversity [65] and genetic distances (F_ST_ distances by Reynolds) were calculated on the basis of haplogroup frequencies (Table S4) using Arlequin 3.11 [66].

In addition, the distance matrix was calculated between the populations studied and those existing in the literature by means of Arlequin 3.11 [66] (Table S4). This distance matrix has been depicted in two dimensions by means of a Multidimensional Scaling (MDS) analysis (SPSS 17 Software) (Figure 2). Furthermore, a Median-Joining Network has been constructed using the 175 sequences of hunter-gatherer and farming individuals that have so far been published (including the samples in this study), using the Network 4.6.0.0 program (www.fluxus-engineering.com) (Figure 3).

## Supporting Information

Table S1 **Summary of results of quantification, replication and cloning from the samples analyzed in the present study (see nomenclature in** **Table 1** **and** **Table 2** **).**
(DOC)

Table S2 **Mitochondrial DNA sequences of the clones from the samples analyzed in the present study (.xls).**
(XLS)

Table S3 **Mitochondrial haplotypes (HVR-I and HVR-II) of researchers and archaeologists that handled the samples analyzed in the present study.**
(DOC)

Table S4 **Prehistoric and present populations compiled from literature that constitutes the database of HVR-I sequences of mtDNA for the present study.** Abbreviations of the populations (Abbrev), number of individuals analyzed (N), number of haplotypes (HT), number of polymorphic sites (s), haplotype diversity (Hd), Variance of Hd, standard deviation of Hd (sd Hd), average number of nucleotide differences (k), Haplogroup (HG) and gene diversity calculated from the frequencies of mitochondrial haplogroups. See abbreviations in Figure 2; * see chronology in Table 1.
(DOC)

Table S5 **Results of F_ST_ analysis.** F_ST_ values (upper the diagonal) and *p*-values with standard deviation (p±de) (under the diagonal) based on haplogroup frequencies (P<0.0001, in gray). See abbreviations in Figure 2.
(XLS)

Table S6 **Primer sequences and annealing temperatures used to amplify the HVR-II of mtDNA, in the present study.**
(DOC)
