# Correction: Ancient DNA from Hunter-Gatherer and Farmer Groups from Northern Spain Supports a Random Dispersion Model for the Neolithic Expansion into Europe

**DOI:** 10.1371/annotation/3dac0b4f-f76e-4bc1-8559-acb41b87b02c

**Published:** 2012-07-10

**Authors:** Montserrat Hervella, Neskuts Izagirre, Santos Alonso, Rosa Fregel, Antonio Alonso, Vicente M. Cabrera, Concepción de la Rúa

There is information missing from the Funding section. The complete Funding statement is: "This work was partly supported by grants CGL-2004-03300, CGL-2007-65515, and a FPI pre-doctoral fellowship for MH (BES-2005-6925) from the Spanish Ministry of Science and Innovation; grant IT542-10 from the Basque Government for Research Groups in the Basque University System, and UFI 11/09 from the University of the Basque Country UPV/EHU. No additional external funding was received for this study. The funders had no role in study design, data collection and analysis, decision to publish, or preparation of the manuscript." 

