# Supplementary material for: Ancient DNA from Hunter-Gatherer and Farmer Groups from Northern Spain Supports a Random Dispersion Model for the Neolithic Expansion into Europe
Source: PLoS One. 2012 Apr 25;7(4):e34417. doi: 10.1371/journal.pone.0034417 (PMC3340892; doi:10.1371/journal.pone.0034417)
Supplement: Table S1 — Summary of results of quantification, replication and cloning from the samples analyzed in the present study (see nomenclature in Table 1 and Table 2 ). (DOC) [file pone.0034417.s001.doc]

**Table S1**. Summary of results of quantification, replication and cloning from the samples analyzed in the present study (see nomenclature in Table 1 and Table 2).

| **Sample** | **HVS-I** | **nt73** | **HVS-II** | **HT** | **HG** | **Nº. molec/μl** | **Duplication** | **Replication by** | **Fragment cloned** |
| --- | --- | --- | --- | --- | --- | --- | --- | --- | --- |
| CAS-21 | rCRS | G | N.A | ht1 | H | 116.5 | UPV/EHU | La Laguna | 2 |
| CAS-33 | rCRS | A | N.A | ht2 | H | 35.5 | NA | NA | 1 |
| CAS-48 | rCRS | G | 73-146-263-285-309.1-310.1-312-313 | ht3 | H | 76 | UPV/EHU | La Laguna | 1 |
| CAS-70 | rCRS | G | 73-146-263-309.1-315.1-320 | ht4 | U | 35.5 | UPV/EHU | N.A | 1 |
| CAS-90 | rCRS | G | 73-146-263-285-309.1-310.1-312-313 | ht3 | H | 411.5 | UPV/EHU | N.A | 1 |
| CAS-148 | 16278- 16311 | G | N.A | ht5 | U | N.A | N.A | N.A | N.A |
| CAS-173 | 16129 | A | N.A | ht6 | H | N.A | UPV/EHU | N.A | 1 |
| CAS-179 | 16069-16126-16195 | G | N.A | ht7 | J | 473.37 | UPV/EHU | N.A | 2 |
| CAS-180 | 16126-16294-16296 | G | N.A | ht8 | T2 | 388.8 | UPV/EHU | N.A | 3 |
| CAS-181 | 16224-16311 | G | 73-263-315.1 | ht9 | K | 388.8 | UPV/EHU | La Laguna | 2 |
| CAS-182 | rCRS | A | N.A | ht2 | H | N.A | UPV/EHU | La Laguna | 3 |
| CAS-183 | 16163 | G | N.A | ht10 | U | 185.2 | N.A | N.A | N.A |
| CAS-191 | 16224-16311 | G | 73-263-315.1 | ht9 | K | 656.3 | UPV/EHU | La Laguna | 2 |
| CAS-193S | 16311 | A | N.A | ht11 | H | 91.03 | UPV/EHU | La Laguna | 1 |
| CAS-194 | 16311 | A | N.A | ht11 | H | 1,167.3 | UPV/EHU | La Laguna | 2 |
| CAS-196 | rCRS | G | 73-146-263-285-309.1-310.1-312-313 | ht3 | H | 411.5 | UPV/EHU | La Laguna | 1 |
| CAS-202 | 16224-16311 | G | 73-263-315.1 | ht9 | K | 524.5 | UPV/EHU | La Laguna | 2 |
| CAS-203 | 16069-16126 | G | N.A | ht12 | J | 176.2 | UPV/EHU | N.A | 1 |
| CAS-204 | 16270-16311 | G | N.A | ht13 | U5 | 458.3 | UPV/EHU | N.A | 2 |
| CAS-216 | rCRS | G | 73-146-263-309.1-315.1-320 | ht4 | U | 495.7 | UPV/EHU | La Laguna | 3 |
| CAS-222 | 16129 | A | N.A | ht6 | H | 975.1 | UPV/EHU | La Laguna | 3 |
| CAS-254 | rCRS | G | 73-146-263-309.1-315.1-320 | ht4 | U | 909.9 | UPV/EHU | N.A | 1 |
| CAS-257 | 16183-16189-16223-16278 | G | N.A | ht14 | X | N.A | N.A | N.A | N.A |
| CAS-258 | rCRS | G | N.A | ht4 | U | 185.1 | UPV/EHU | N.A | 3 |
| CAS-341 | 16129 | A | N.A | ht6 | H | 820.9 | N.A | N.A | 2 |
| CAS-497 | rCRS | A | N.A | ht2 | H | 479.6 | UPV/EHU | N.A | 1 |
| CAS-517 | 16365 | N.A | N.A | ht15 | U | N.A | N.A | N.A | N.A |
| PAT-1E1 | rCRS | G | N.A | ht4 | U | 122.9 | UPV/EHU | La Laguna | 1 |
| PAT-1E3 | rCRS | A | N.A | ht2 | H | 333.5 | UPV/EHU | La Laguna | 1 |
| PAT-1E4 | 16209 | A | N.A | ht16 | H3 | N.A | UPV/EHU | N.A | 1 |
| PAT-1E5 | 16092-16311 | A | N.A | ht17 | H | N.A | UPV/EHU | N.A | 2 |
| PAT-2E1 | rCRS | A | N.A | ht2 | H | 751.7 | UPV/EHU | La Laguna | 5 |
| PAT-2E2 | 16092-16224-16311 | G | 73-146-263-315.1C | ht18 | K | 258.5 | UPV/EHU | N.A | 2 |
| PAT-3E2 | 16311 | A | N.A | ht19 | HV | 287.3 | N.A | N.A | 3 |
| PAT-4E1 | 16129-16223 | G | N.A | ht20 | I | 22.8 | UPV/EHU | N.A | 5 |
| PAT-4E2 | 16209 | A | N.A | ht16 | H3 | 372.4 | UPV/EHU | N.A | 5 |
| ERR-1 | 16270 | G | N.A | ht21 | U5 | 6,666.7 | N.A | La Laguna | 1 |
| MZ-1 | 16270 | G | 73-263-315.1C | ht21 | U5 | 766.7 | N.A | La Laguna | 1 |
| FH-1 | rCRS | G | N.A | ht4 | U | 1,233.3 | N.A | La Laguna | N.A |
| FH-2 | 16192-16270 | G | N.A | ht22 | U5a | 496.7 | N.A | La Laguna | N.A |
| FH-3 | 16093 | N.A | N.A | ht23 | H | N.A | N.A | N.A | N.A |
| FH-4 | rCRS | N.A | N.A | ht4 | U | N.A | N.A | N.A | N.A |
| FH-5 | rCRS | N.A | N.A | ht4 | U | N.A | N.A | N.A | N.A |
| FH-6 | rCRS | A | N.A | ht2 | H | 554.5 | N.A | La Laguna | N.A |
| URT-1 | rCRS | A | N.A | ht2 | H | 3,333.33 | N.A | La Laguna | N.A |
| URT-2 | 16192-16270 | G | N.A | ht22 | U5a | 1,420.33 | N.A | La Laguna | N.A |
| CH-1 | 16093-16362 | G | N.A | ht24 | H6 | 669.21 | N.A | N.A | N.A |
| PS-1 | rCRS | A | N.A | ht2 | H | 1,229.6 | N.A | N.A | N.A |
| AIZ | 16051-16093*-16189-16192-16270 | G | N.A | ht25 | U5b1 | 12,797.0 | UPV/EHU | INTCF Madrid | 6 |

rCRS: revised Cambridge Reference Sequence

N.A.: Not analysed

* Heteroplasmic position
