# Supplementary material for: Ancient DNA from Hunter-Gatherer and Farmer Groups from Northern Spain Supports a Random Dispersion Model for the Neolithic Expansion into Europe
Source: PLoS One. 2012 Apr 25;7(4):e34417. doi: 10.1371/journal.pone.0034417 (PMC3340892; doi:10.1371/journal.pone.0034417)
Supplement: Table S3 — Mitochondrial haplotypes (HVR-I and HVR-II) of researchers and archaeologists that handled the samples analyzed in the present study. (DOC) [file pone.0034417.s003.doc]

**Table S3.** Mitochondrial haplotypes (HVR-I and HVR-II) of researchers and archaeologists that handled the samples analyzed in the present study.

| **Lab** | **Researcher** | **HVR I haplotype** | **HVR II haplotype** |
| --- | --- | --- | --- |
| Univ. Basque Country (Spain) |  |  |  |
|  | Researcher #1 | rCRS | 263G-309.1C -315.1C |
|  | Researcher #2 | rCRS | 150T-152C-263G-315.1C |
|  | Researcher #3 | 189 | 263G-315.1C |
|  | Researcher #4 | 319 | 263G-315.1C |
|  | Researcher #5 | 092-224-311 | 73G-146C-263G-315.1C |
| Univ. La Laguna (Spain) |  |  |  |
|  | Researcher #1 | 129-316 | N.A |
|  | Researcher #2 | 223-278-311-362 | N.A |
| Archaeologists |  |  |  |
|  | Researcher #1 | 304 | N.A |
|  | Researcher #2 | 291 | N.A |
|  | Researcher #3 | 220 | N.A |
|  | Researcher #4 | 051-162-264 | N.A |
|  | Researcher #5 | 298 | N.A |

HVR-I: Hypervariable Region I of mtDNA. rCRS: revised Cambridge Reference Sequence. The figures correspond to the position in region I of HVR of mt DNA that changes with respect to the rCRS. Precise mitochondrial coordinates can be obtained by adding 16.000.
