# Supplementary material for: Ancient DNA from Hunter-Gatherer and Farmer Groups from Northern Spain Supports a Random Dispersion Model for the Neolithic Expansion into Europe
Source: PLoS One. 2012 Apr 25;7(4):e34417. doi: 10.1371/journal.pone.0034417 (PMC3340892; doi:10.1371/journal.pone.0034417)
Supplement: Table S4 — Prehistoric and present populations compiled from literature that constitutes the database of HVR-I sequences of mtDNA for the present study. Abbreviations of the populations (Abbrev), number of individuals analyzed (N), number of haplotypes (HT), number of polymorphic sites (s), haplotype diversity (Hd), Variance of Hd, standard deviation of Hd (sd Hd), average number of nucleotide differences (k), Haplogroup (HG) and gene diversity calculated from the frequencies of mitochondrial haplogroups. See abbreviations in Figure 2; * see chronology in Table 1. (DOC) [file pone.0034417.s004.doc]

**Table S4.** **Table S4.** Prehistoric and present populations compiled from literature that constitutes the database of HVR-I sequences of mtDNA for the present study. Abbreviations of the populations (Abbrev), number of individuals analyzed (N), number of haplotypes (HT), number of polymorphic sites (s), haplotype diversity (Hd), Variance of Hd, standard deviation of Hd (sd Hd), average number of nucleotide differences (k), Haplogroup (HG) and gene diversity calculated from the frequencies of mitochondrial haplogroups. See abbreviations in Figure 2; * see chronology in Table 1.

| **Abbrev,** | **Location** | **Chronology** | **N** | **HT** | **S** | **Hd** | **Variance**  **Hd** | **sd Hd** | **K** | **HG** | **Gene Diversity** | **References** |
| --- | --- | --- | --- | --- | --- | --- | --- | --- | --- | --- | --- | --- |
| HG_SCA | Scandinavia | 6,000 BP | 19 | 14 | 16 | 0,95 | 0,00082 | 0,029 | 3.082 | 4 | 0,2982±0,1239 | [1] |
| HG_CE | Central and Eastern Europe | 11,000 BP | 22 | 14 | 19 | 0,95 | 0,00106 | 0,033 | 3,082 | 2 | 0,3333±0,1293 | [2] |
| HG_CANT | Cantabrian Fringe of Spain | several sites* | 4 | 4 | 6 | 1 | 0,03125 | 0,177 | 3.167 | 3 | 0,6667±0,2041 | Present study |
| NEO_NAVARRE | Navarre, Spain | several sites* | 36 | 16 | 19 | 0,84 | 0,00318 | 0,056 | 2,019 | 8 | 0,7143±0,1239 | Present study |
| NEO_FR | France | 3,000 BP | 29 | 14 | 20 | 0,91 | 0,00102 | 0,032 | 3.557 | 7 | 0,8000±0,1217 | [3] |
| NEO_CAT | East Spain | 5,500 BP | 11 | 8 | 18 | 0,93 | 0,00442 | 0,066 | 4.582 | 6 | 0,8545±0,0852 | [4] |
| NEO_CE | Central and Eastern Europe | 7,500 BP | 42 | 24 | 31 | 0,97 | 0,00014 | 0,012 | 5,102 | 9 | 0,8792±0,0226 | [5, 6] |
| SJaPL | Alava, Spain | 5,000 BP | 61 | NA | NA | NA | NA | NA | NA | 5 | 0,7662±0,0466 | [7] |
| Longar | Navarre, Spain | 4,500 BP | 27 | NA | NA | NA | NA | NA | NA | 4 | 0,7138±0,0593 | [7] |
| Pico Ramos | Bizkaia, Spain | 4,500 BP | 24 | NA | NA | NA | NA | NA | NA | 5 | 0,7935±0,0494 | [7] |
| Aldaieta | Alava, Basque Country | VI-VII c. AD | 63 | 17 | 31 | 0.548 | 0,0087 | 0.0145 | 1,6129 | 7 | 0,7292±0,0824 | [8-10] |

| **Abbrev.** | **Populations** | **N** | **HG** | **Gene diversity** | **References** |
| --- | --- | --- | --- | --- | --- |
| SE | Bulgaria |  |  |  |  |
|  | Romania | 233 | 9 | 0.7719±0.091 | [11,12] |
| MdE | Greece |  |  |  |  |
|  | Albania | 107 | 9 | 0.7458±0.0263 | [12,13] |
| MdC | Italy |  |  |  |  |
|  | Rome |  |  |  |  |
|  | Sicily |  |  |  |  |
|  | Sardinia | 214 | 9 | 0.7488±0.0196 | [4,12,15,16] |
| Basques | Gipuzkoa |  |  |  |  |
|  | Bizkaia |  |  |  |  |
|  | Álava |  |  |  |  |
|  | Basque Country | 407 | 9 | 0.7558±0.0120 | [12, 17-20] |
|  | Galicia |  |  |  |  |
| Can_F | Asturias |  |  |  |  |
|  | Cantabria | 372 |  |  | [19-22] |
| Alps | Switzerland |  |  |  | [12,23] |
|  | Germany South | 218 | 9 | 0.7215±0.0249 |  |
|  | Poland |  |  |  |  |
|  | Czechoslovakia |  |  |  |  |
|  | Germany |  |  |  |  |
| NC | Denmark | 332 | 9 | 0.7193±0.0134 | [12,23,24] |
|  | Switzerland | 478 |  |  |  |
| SCA | Norway |  | 9 | 0.6664±0.0108 | [12, 25] |
| NW | France |  |  |  |  |
|  | England |  |  |  |  |
|  | Scotland | 361 | 9 | 0.7402±0.0160 | [12, 26-27] |
| NE | Russia |  |  |  |  |
|  | Estonia |  |  |  |  |
|  | Czech Republic | 544 | 9 | 0.7390±0.0106 | [12, 28-31] |
| Ib_Pen | Andalusia |  |  |  |  |
|  | Castilla-León |  |  |  |  |
|  | Portugal |  |  |  |  |
|  | Catalonia | 627 | 8 | 0.6711±0.0118 | [18,32-35] |
| N Caucasus | Northern Caucasus | 208 | 9 | 0.7871±0.0147 | [12, 22] |
| Iraq |  | 116 | 6 | 0.8111±0.0130 | [12] |
| Syria |  | 69 | 8 | 0.8250±0.0233 | [12] |
| Palestine |  | 117 | 7 | 0.8131±0.0158 | [12,16] |
| Turkey |  | 218 | 9 | 0.8169±0.0126 | [11,12,36] |
| Kurdistan |  | 53 | 8 | 0.8512±0.0209 | [12] |
| Armenia |  | 191 | 8 | 0.7934±0.0153 | [12] |

**References**

1.Malmström H, Gilbert MT, Thomas MG, Brandstrom M, Stora J, et al. (2009) Ancient DNA reveals lack of continuity between neolithic hunter-gatherers and contemporary Scandinavians. Curr Biol 19: 1758-1762

2. Bramanti B, Thomas MG, Haak W, Unterlaender M, Jores P, et al. (2009) Genetic discontinuity between local hunter-gatherers and Central Europe's first farmers. Science 326*:* 137-140

3. Lacan M, Keyser C, Ricaut FX, [Brucato N](http://www.ncbi.nlm.nih.gov/pubmed?term="Brucato N"%5BAuthor%5D), [Duranthon F](http://www.ncbi.nlm.nih.gov/pubmed?term="Duranthon F"%5BAuthor%5D), et al.(2011) Ancient DNA reveals male diffusion through the Neolithic Mediterranean route. Pro Natl Acad Sci U S A 108: 9788-9791.

4. Sampietro ML, Lao O, Caramelli DLM, Pou R, Marti M, Bertranpetit J, et al. (2007) Palaeogenetic evidence supports a dual model of Neolithic spreading into Europe. Proc Biol Sci 274: 2161-67.

5. Haak W, Forster P, Bramanti B, Matsumura S, Brandt G, et al. (2005) Ancient DNA from the first European farmers in 7500-year-old Neolithic sites. Science 310: 1016-18.

6. Haak W, Balanovsky O, Sanchez JJ, [Koshel S](http://www.ncbi.nlm.nih.gov/pubmed?term="Koshel S"%5BAuthor%5D), [Zaporozhchenko, V](http://www.ncbi.nlm.nih.gov/pubmed?term="Zaporozhchenko V"%5BAuthor%5D)., [Adler, C.J](http://www.ncbi.nlm.nih.gov/pubmed?term="Adler CJ"%5BAuthor%5D), et al. (2010) Ancient DNA from European early neolithic farmers reveals their near eastern affinities. PLoS Biol 8: e1000536.

7. Izagirre N, de la Rúa C (1999) An mtDNA analysis in ancient Basque populations: implications for haplogroup V as a marker for a major paleolithic expansion from southwestern Europe. Am J Hum Genet 65: 199-207.

8. Alzualde A, Izagirre N, Alonso S, Alonso A, de la Rua C (2005) Temporal mitochondrial DNA variation in the Basque Country: influence of post-neolithic events. Ann Hum Genet 69: 665-79.

9. Alzualde A, Izagirre N, Alonso S, Alonso A, et al. (2006) Insights into the "isolation" of the Basques: mtDNA lineages from the historical site of Aldaieta (6th-7th centuries AD). Am J Phys Anthropol 130: 394-404.

10. Alzualde A, Izagirre N, Alonso S, Alonso A, Azkarate A, et al. (2007) Influences of the European Kingdoms of Late Antiquity on the Basque Country: An Ancient DNA Study. Curr Anthropol 48: 155-62.

11. Calafell F, Underhill P, Tolun A, Angelicheva D, Kalaydjieva L (1996) From Asia to Europe: mitochondrial DNA sequence variability in Bulgarians and Turks. Ann Hum Genet 60: 35-49.

12. Richards M, Macaulay V, Hickey E, Vega E, Sykes B, et al. (2000) Tracing European founder lineages in the Near Eastern mtDNA pool. Am J Hum Genet 67: 1251-76.

13. Belledi M, Poloni ES., Casalotti R, Conterio F, Mikerezi I, et al. (2000) Maternal and paternal lineages in Albania and the genetic structure of Indo-European populations. Eur J Hum Genet 8: 480-486.

14. Francalacci P, Bertranpetit J, Calafell F, Underhill PA (1996) Sequence diversity of the control region of mitocnondrial DNA in Tuscany and its implications for the peopling of Europe. Am J Phys Anthropol 100: 443-460.

15. Torroni A, Bandelt HJ, D'Urbano L, Lahermo P, Moral P, et al. (1998) mtDNA analysis reveals a major late Paleolithic population expansion from southwestern to northeastern Europe. Am J Hum Genet 62: 1137-52.

16. Di Rienzo A, Wilson, AC (1991) Branching pattern in the evolutionary tree for human mitochondrial DNA. Proc Natl Acad Sci USA 88: 1597-1601.

17. Bertranpetit J, Sala J, Calafell F, Underhill PA, Moral P, et al. (1995) Human mitochondrial DNA variation and the origin of Basques. Ann Hum Genet 59: 63-81.

18. Corte-Real HB, Macaulay VA, Richards MB, Hariti G, Issad MS, et al. (1996) Genetic diversity in the Iberian Peninsula determined from mitochondrial sequence analysis. Ann Hum Genet 60: 331-350.

19. Alfonso-Sánchez MA, Cardoso S, Martínez-Bouzas C, Peña JA, Herrera, RJ, et al. (2008) Mitochondrial DNA haplogroup diversity in Basques: A reassessment based on HVI and HVII polymorphisms. Am J Hum Biol 20: 154-156.

20. García O, Fregel R, Larruga JM, Fregel R, Larruga JM, et al. (2011) Using mitochondrial DNA to test the hypothesis of a European post-glacial human recolonization from the Franco-Cantabrian refuge. Heredity 106: 37-45.

21. Salas A, Comas D, Lareu MV, Bertranpetit J, Carracedo A (1998) mtDNA analysis of the Galician population: a genetic edge of European variation. Eur J Hum Genet 6: 365-75.

22. Maca-Meyer N, Gonzalez AM, Pestano J, Flores C, Larruga JM, et al. (2003) Mitochondrial DNA transit between West Asia and North Africa inferred from U6 phylogeography BMC Genet 4: 15.

23. Pult I, Sajantila A, Simanainen J, Georgiev O, Schaffner W, et al. (1994) Mitochondrial DNA sequences from Switzerland reveal striking homogeneity of European populations. Biol Chem Hoppe Seyler 375: 837-840.

24. Poetsch M, Wittig H, Krause D, Lignitz E (2003) Mitochondrial diversity of a northeast German population sample. Forensic Sci Int 137: 125-132.

25. Opdal SH, Rognum TO, Vege A, Stave AK, Dupuy BM, et al. (1998) Increased number of substitutions in the D-loop of mitochondrial DNA in the sudden infant death syndrome. Acta Paediatr 87: 1039-1044.

26. Helgason A, Sigurethardottir S, Gulcher JR, Ward R, et al. (2000) mtDNA and the origin of the Icelanders: deciphering signals of recent population history. Am J Hum Genet 66: 999-1016.

27. Piercy R, Sullivan KM, Benson N, Gill, P (1993) The application of mitochondrial DNA typing to the study of white Caucasian genetic identification. Int J Legal Med 106: 85-90.

28. Malyarchuck, B A, Grzybowski, T, Derenko, MV, Czarny, J, Wozniak, et al. (2003) Mitochondrial DNA variability in Poles and Russians. Ann Hum Genet 66: 261-83.

29. Malyarchuk BA, Perkova MA, Derenko MV, Vanecek T, Lazur J, et al. (2008) Mitochondrial DNA variability in Slovaks, with application to the Roma origin. Ann Hum Genet 72: 228-40.

30. Sajantila A, Lahermo P, Anttinen T, Lukka M, Sistonen P, et al. (1995) Genes and languages in Europe: an analysis of mitochondrial lineages. Genome Res 5: 42-52.

31. Sajantila A, Salem AH, Savolainen P, Bauer K, Gierig C, et al. (1996) Paternal and maternal DNA lineages reveal a bottleneck in the founding of the Finnish population. Proc Natl Acad Sci U S A 93: 12035-12039.

32. González AM, Brehm A, Pérez JA, Maca-Meyer N, Flores C, et al. (2003) Mitochondrial DNA Affinities at the Atlantic Fringe of Europe. Am J Phys Anthropol 120: 391-404.

33. Alvarez-Iglesias JC, Johnson DL, Lorente JA, Martinez-Espin E, Martinez-Gonzalez LJ, et al. (2007) Characterization of human control region sequences for Spanish individuals in a forensic mtDNA data set. Leg Med (Tokyo) 9: 293-304.

34. Larruga JM, Diez F, Pinto FM, Flores C, Gonzalez AM (2001) Mitochondrial DNA characterisation of European isolates: the Maragatos from Spain. Eur J Hum Genet 9: 708-16.

35. Plaza S, Calafell F, Helal A, Bouzerna N, Lefranc G, et al. (2003) Joining the Pillars of Hercules: mtDNA sequences show multidirectional gene flow in the western Mediterranean. Ann Hum Genet 67: 312-328.

36. Comas D, Calafell F, Mateu E, Perez-Lezaun A, Bertranpetit J (1996) Geographic variation in human mitochondrial DNA control region sequence: the population history of Turkey and its relationship to the European populations. Mol Biol Evol 13: 1067-77.
