# Supplementary material for: Ancient DNA from Hunter-Gatherer and Farmer Groups from Northern Spain Supports a Random Dispersion Model for the Neolithic Expansion into Europe
Source: PLoS One. 2012 Apr 25;7(4):e34417. doi: 10.1371/journal.pone.0034417 (PMC3340892; doi:10.1371/journal.pone.0034417)
Supplement: Table S6 — Primer sequences and annealing temperatures used to amplify the HVR-II of mtDNA, in the present study. (DOC) [file pone.0034417.s006.doc]

**Table S5.** Primer sequences and annealing temperatures used to amplify the HVR-II of mtDNA, in the present study.

| **Primer sequences (5’→3’)** | **Tª (ºC)** | **Size(bp)** |
| --- | --- | --- |
| **7F** TTC CTA CTT CAG GGT CAT AAA GCC | 61 | 115 |
| **7R:** ACC AAA TGC ATG GAG AGC TC |
| **8F** GGT CTA TCA CCC TAT TAA CC | 60 | 121 |
| **8R** GAT ACT GCG ACA TAG GGT GC |
| **9F** TGC ACG CGA TAG CAT TGC GAG | 58 | 136 |
| **9R:**ATT ATG TCC TAC AAG CAT TAA TT |
| **10F** CGC ACC TAC GTT CAA TAT T AC | 57 | 121 |
| **10R** ATG ATG TCT GTG TGG AAA G |
| **11F** TTG AAT GTC TGC ACA GCC | 57 | 148 |
| **11R** TCT GGT TAG GCT GGT GTT AG |
| **12F:** TGG CCA CAG CAC TTA AAC AC | 55 | 109 |
| **12R:** CTG TTA AAA GTG CAT ACC GCC |
